# Pregnancy Coercion as a Risk Factor for HIV and Other Sexually Transmitted Infections Among Young African American Women

**DOI:** 10.1097/QAI.0000000000002174

**Published:** 2019-10-29

**Authors:** Ariadna Capasso, Ralph J. DiClemente, Gina M. Wingood

**Affiliations:** a Social and Behavioral Sciences Department, College of Global Public Health, New York University, New York, NY; and; b Department of Sociomedical Sciences, Mailman School of Public Health, Columbia University, New York, NY.

**Keywords:** human immunodeficiency virus, sexually transmitted infections, pregnancy coercion, reproductive coercion, women, African American

## Abstract

Pregnancy coercion (PC), defined as a restriction of women's reproductive autonomy, may be associated with increased HIV and sexually transmitted infection (STI) risk. However, there are few empirical studies defining the association between PC and HIV risk, particularly among vulnerable African American women.

**Setting and Methods:**

African American women (N = 560), ages 17–24, completed an audio computer-assisted self-interview assessing PC prevalence and its association with HIV/STI risk. Women were screened for prevalent STIs using polymerase chain reaction assays. Multivariate logistic and linear regressions evaluated the association of PC and multiple HIV/STI risk-associated outcomes.

**Results:**

Women who had experienced PC in the last 3 months, relative to those not experiencing PC, were 78% more likely to test positive for an STI [adjusted odds ratio = 1.78, 95% confidence interval (CI) = 1.10 to 2.90]. Among women who experienced PC, odds of noncondom use in their last sexual encounter were 3.45-fold greater relative to women not experiencing PC (95% CI = 1.55 to 7.85). Women who experienced PC had lower condom use intentions (coefficient, −1.31, *P* = 0.002), greater fear of condom negotiation, and perceived more barriers to condom use (coefficients, 3.89 and 5.74, respectively, both *P* < 0.001). Women who experienced PC had 1.98 (95% CI = 1.22 to 3.21) and 1.82 (95% CI = 1.09 to 3.04) odds of depression and HIV worry relative to women not experiencing PC.

**Conclusion:**

Among African American women, PC was associated with a range of adverse sexual health outcomes and HIV/STI-related behaviors and attitudes. The findings underscore the need for promoting gender-equitable social norms in HIV prevention interventions.

## INTRODUCTION

African American women are disproportionately affected by HIV and othiker sexually transmitted infections (STIs). In 2017, 24.9/100,000 African American women were diagnosed with HIV compared with 1.7/100,000 white women,^1^ with heterosexual transmission accounting for 95.2% of new HIV infections.^2^ Similar racial disparities are observed in the distribution of STIs, such as chlamydia, gonorrhea, and trichomoniasis.^3–5^

Noncondom-protected heterosexual contact is associated with acquisition and transmission of HIV and other STIs,^6–8^ with extensive empirical evidence demonstrating a robust association between bacterial, viral, and parasitic STIs and risk of HIV seroconversion.^5,9–12^ A recent meta-analysis concluded that STIs confer a 2- to 3-fold increase in HIV-1 susceptibility.^11^

In addition to biological factors, numerous individual-level cognitive and behavioral factors are associated with increased HIV/STI risk, such as number of sexual partners, depression, and substance use.^13^ However, although there is considerable empirical data describing the association between myriad individual-level determinants and HIV/STIs, one area that remains understudied is the association of relational-level factors to HIV/STI acquisition.

One such relational factor is pregnancy coercion (PC). PC is a form of power and control—often exerted by a male sexual partner—that interferes with a woman's autonomous decisions regarding reproduction, for example by exerting pressure on the woman to become or remain pregnant.^14,15^ Prevalence of PC is higher among African American women than among women of other racial/ethnic groups.^16^ Miller reported lifetime rates of PC of 25.9% among African American women versus 13.3% among white women.^14^

PC has often been examined in the context of intimate partner violence with a focus on unintended pregnancy as an outcome^16,17^; however, PC as a risk factor for HIV/STIs, particularly among women who drink regularly, has not been studied. The association of intimate partner violence and sexual risk behaviors has been documented.^18^ However, the extent to which PC affects sexual behavior is an important and understudied area of research. Given the evidence gap with respect to PC and HIV/STI risk behaviors among African American women, the objective of this paper is to examine the association between PC and multiple adverse biological and psychosocial factors associated with HIV/STI risk in this population.

## METHODS

### Data

We conducted secondary analysis of baseline data collected from January 2012 to February 2014 as part of a randomized controlled trial to assess the efficacy of an HIV prevention intervention among young African American women who consumed alcohol. The methods have been described in detail elsewhere.^19,20^ Briefly, 560 African American women, aged 17–24, who reported at least 3 occasions of alcohol consumption and at least one instance of unprotected vaginal sex in the past 90 days, were recruited through street intercept and community outreach in Atlanta, GA. At baseline, all participants completed an audio computer-assisted self-interview and provided 2 vaginal swab specimens, which were assayed for 3 common STIs: chlamydia, gonorrhea, and trichomoniasis. All participants who tested STI-positive were treated with a single-dose antimicrobial and provided risk-reduction counseling. Written informed consent was obtained from all participants. The trial was registered in ClinicalTrials.gov (NCT01553682); Emory University's institutional review board approved all study procedures.

### Outcome Measures

#### Biological Outcomes

Participants' self-collected vaginal swabs (Swube applicator; Becton Dickinson Microbiology Systems, Sparks, MD) were evaluated for *Chlamydia trachomatis* and *Neisseria gonorrhoeae* using BD ProbeTec ET GC/CT-Amplified DNA assays (Becton, Dickinson and Company)^21^ and for *Trichomonas vaginalis* using TaqMan polymerase chain reaction assay (Thermo Fisher Scientific, Waltham, MA). For these analyses, we created a composite variable, whereby women who tested positive for one or more STIs were coded as “1” and those who tested negative for all 3 were coded as “0.”

In addition, we included 2 questions related to pregnancy history as proxies for occurrence of unprotected sex: ever having been pregnant (0 = No; 1 = Yes) and ever having an abortion or miscarriage (0 = No; 1 = Yes).

#### Behavioral Outcomes

Sexual behaviors were assessed by 2 measures: number of sex partners in the past 3 months (dichotomized as 1 partner; or = >2 partners); and condomless sex in the last sexual encounter, coded as No or Yes.

#### Condom Use Attitudes

Intention to purchase, discuss with a partner, and use condoms was measured with a 4-item scale.^22^ The scale included questions such as *“How likely is it that you will use a condom every time you have sexual intercourse in the next 3 months?”* with answer choices from 1 (will not happen) to 4 (will definitively happen). This scale yielded a range of 4–16; higher scores indicate higher condom use intentions. Cronbach alpha was 0.88.

Fear of discussing or negotiating condom use with a sex partner was assessed with a 7-item scale,^23^ with total scores ranging from 7 (never fearful) to 35 (always fearful). Sample questions included, *“I have been worried that if I talked about using condoms with my boyfriend or sex partner he would hit, push, or kick me”* and *“I have been worried that if I talked about using condoms with my boyfriend or sex partner he would leave me.”* Cronbach alpha was 0.87.

Perceived sex partner barriers to condom use were measured by a 6-item scale.^24^ The scale consisted of items such as *“If I asked my partner to use a condom, he might think I was cheating on him.”* Each item was measured on a 5-point Likert scale with respondent options ranging from 1 (strongly disagree) to 5 (strongly agree). The possible total score ranged from 6 to 30; with higher scores indicative of higher perceived partner resistance to condom use. The scale had high internal consistency reliability (α = 0.92).

#### Psychological Outcomes

Given the association between depression and sexual risk behaviors,^25^ depression was included in this analysis. Depressive symptoms were measured using the Revised Center for Epidemiologic Studies Depression Scale, which has been found to have strong external validity.^26^ Following Melchior, a cut-off point of 7 was used to dichotomize the variable as women without and with depressive symptomatology (coded as 0 and 1, respectively). Cronbach alpha was α = 0.91.

We also included one item to assess women's perceptions of their risk level. Response options to the question, *“How much do you worry that you could get HIV?”* ranged from 1 (not worried) to 5 (worried a lot). The variable was dichotomized as: 0 = No or little worry; and 1 = Some to a lot of worry.

#### Interpersonal Outcomes

Abusive relationships are indicative of gender-based power imbalances, often associated with forced unprotected sex.^27,28^ The association of intimate partner violence and both reproductive coercion and risk of HIV/STI acquisition has been documented.^14,29–31^ Therefore, we included abuse variables in our analysis to control for this effect. Recent intimate partner violence was assessed by 2 questions, each analyzed separately, and representing 2 types of abuse frequently reported in the literature: physical and sexual abuse.^32^ For example, physical abuse was assessed with the Yes/No question, *“In the past 3 months, have you been physically abused by your boyfriend? (hit, punched, kicked, slapped, etc.)?”*

Because of its linkage to PC, birth control sabotage was also included.^16^ The construct was measured using the Miller scale,^14^ consisting of 5 binary (Yes/No) items. An example was, *“In the past 3 months, has someone you were dating or going out with ever made you have sex without a condom so you would get pregnant?”* The variable was coded as a dichotomous variable, with no experiences of birth control sabotage coded as 0 and at least one experience coded as 1. Internal consistency reliability was high (α = 0.94).

#### Demographic Measures

We controlled for 3 socioeconomic variables in the analysis: age; education (0 = Did not complete high school; 1 = Completed high school/equivalence degree or above); and main source of income (0 = Job, family, or public assistance; 1 = Boyfriend or partner).

#### Independent Measure

The main independent measure was experience of PC by a male sex partner in the past 3 months. PC was assessed by 4 items from the Miller validated scale.^14^ Binary (Yes/No) questions included items such as *“In the past 3 months, has someone you were dating or going out with ever said he would leave you if you did not get pregnant?”* and *“In the past 3 months, has someone you were dating or going out with ever told you he would have a baby with someone else if you did not get pregnant?”* Respondents who answered *“No”* to all items were coded as 0; respondents who answered *“Yes”* to at least one item were coded as 1. The scale had high internal reliability (α = 0.94).

### Statistical Analyses

Descriptive statistics were used to summarize socioeconomic, biological, behavioral–cognitive, and interpersonal outcomes associated with HIV/STI risk. Differences in outcome measures by experience of PC were assessed using two-sided Wilcoxon rank sum tests for non-normally distributed continuous variables and Pearson χ^2^ analyses for categorical variables. All variables with a significance level below 0.2 in bivariate analysis were tested in multilevel regression models. Linear and logistic regression models determined whether PC was independently and significantly associated with HIV/STI infection, while controlling for other important sociodemographic variables, including: age, income source, and educational attainment. Relevant interactions were also tested. A *P*-value of 0.05 or less was considered significant. Logistic models were tested for goodness-of-fit using the Hosmer–Lemeshow test. Relevant model assumptions were tested in postestimation analyses. All analyses were conducted using Stata 15.1 (Stata Corporation LP, College Station, TX).

Based on model assumption testing some potential outliers were dropped from the model testing. Four variables had missing data or were missing more than 0.5% of data points. The sexual and physical abuse questions were only asked of women who were currently in a relationship (n = 350). There were 210 (37.5%) missing data points for the questions related to having a miscarriage or an abortion. We created dummy variables to test for differences in the distribution of PC among those with and without missing data in these 4 variables. No statistically significant differences were observed.

## RESULTS

Median age was 20 years (range 17–24), with 185 (33.0%) participants not having completed high school. A total of 189 (33.8%) women tested positive for any STI at baseline. Among all participants, 128 (22.9%) reported ever experiencing PC, and 81 (14.5%) had done so in the last 3 months. More than 3-quarters of the sample engaged in condomless sex (76.5%) and more than half worried about acquiring HIV (58.4%) (Table 1).

**TABLE 1.**
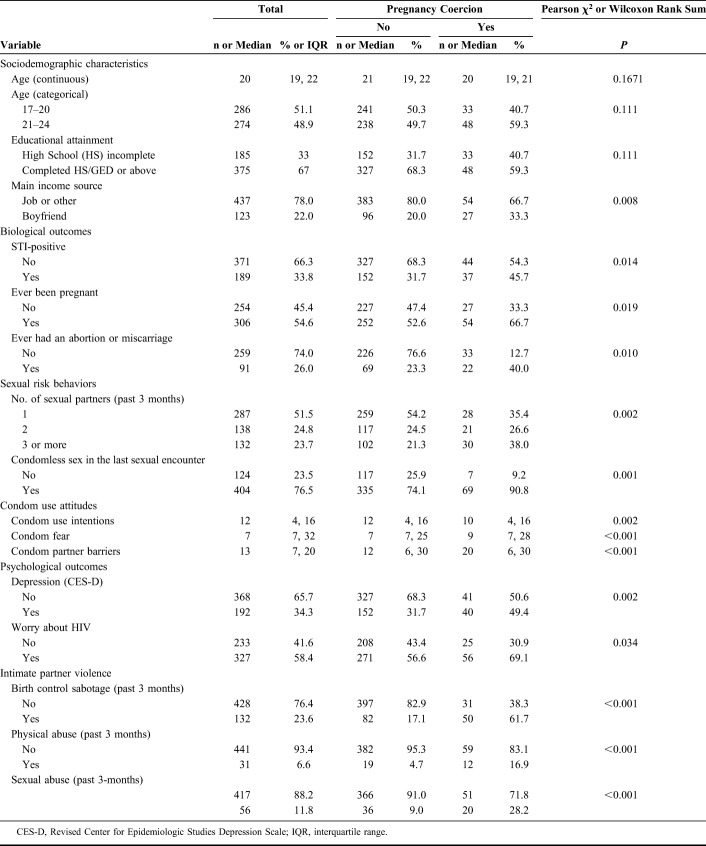
Characteristics of 560 African American Women by Past 3-Month Experience of Pregnancy Coercion, Atlanta, GA, January 2012 to February 2014

When controlling for age, educational attainment, and main income source, women who experienced PC in the last 3 months were 78% more likely to test positive for an STI than those who did not [adjusted odds ratio (AOR) = 1.78, 95% confidence interval (CI) = 1.10 to 2.90]. In addition, they were 89% (AOR = 1.89, 95% CI = 1.12 to 3.18) and 126% (AOR = 2.26, 95% CI = 1.21 to 4.24) more likely to have ever been pregnant or had an abortion or miscarriage, respectively. Results are shown in Table 2.

**TABLE 2.**
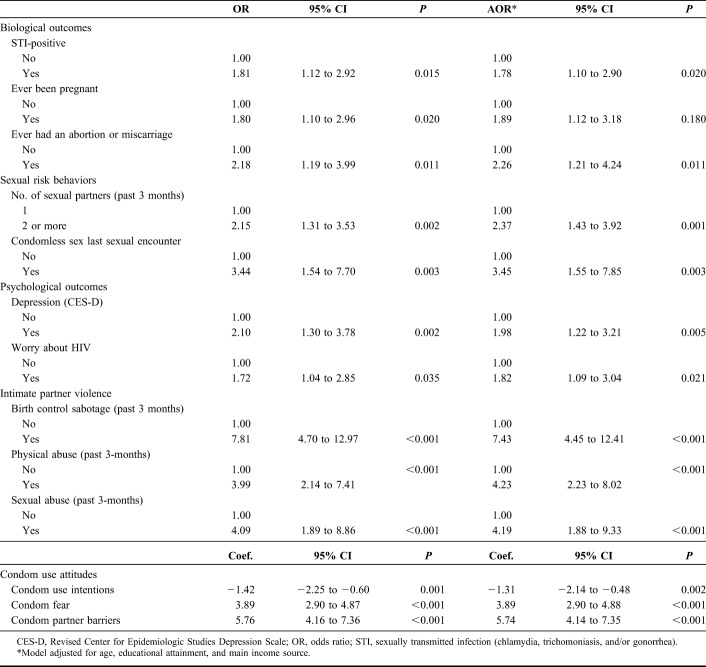
Logistic and Linear Regressions of Past 3-Month Pregnancy Coercion and HIV-Associated Outcomes Among 560 African American Women in Atlanta, GA, January 2012 to February 2014

Among women who experienced PC in the last 3 months, odds of having 2 or more sex partners in the past 3 months were 2.37 (95% CI = 1.43 to 3.92) and of not using a condom in the last sexual encounter were 3.45 (95% CI = 1.55 to 7.85) those of women not experiencing coercion, respectively.

Women who were coerced had lower intentions of purchasing or using a condom than those who did not experience coercion (coefficient, −1.31, *P* = 0.002). In addition, women who were coerced by sex partners had more fear of negotiating condom use and perceived more barriers to using a condom than those who did not (coefficients, 3.89 and 5.74, respectively, both *P* < 0.001).

Women who experienced PC in the past 3 months were almost twice as likely to have symptoms consistent with depression (AOR = 1.98, 95% CI = 1.22 to 3.21) than those who did not. In addition, they were more likely to worry about acquiring HIV (AOR = 1.82, 95% CI = 1.09 to 3.04).

PC was closely associated with birth control sabotage and intimate partner violence. Odds of experiencing birth control sabotage, physical abuse, and sexual abuse among women who were coerced to get pregnant, relative to women not coerced, were 7.43 (95% CI = 4.45 to 12.41), 4.23 (95% CI = 2.23 to 8.02), and 4.19 (95% CI = 1.88 to 9.33), respectively. None of the tested interactions were significant (data not shown).

## DISCUSSION

This paper contributes to the evidence that PC is a salient risk factor for acquisition of HIV and other STIs. Although young African American women engage less often in sexual risk behaviors than women of other race/ethnicities,^33^ rates of HIV/STIs are markedly higher in this population. Pressure by male partners to get pregnant could be a potential driver of risk behaviors associated with higher infection rates.

Prevalence of STIs was high in our study, with more than 1 in 3 (33.8%) women testing positive for at least one infection. These rates are higher than those reported in other samples of African American women (17.0%–29.3%).^31,33^ More than 1 in 5 women in our study experienced PC. The lifetime prevalence of PC observed in this sample (22.9%) was similar to that reported in previous studies of African American women (21.0%–25.9%),^14,34^ but higher than in other racial/ethnic groups (6.8%).^35^ The past 3-month prevalence of PC was 14.5%, higher than that reported among African American women in other samples (9.3%–12.5%)^17^ and strikingly higher than rates reported for other racial/ethnic groups (5.1%).^17^

The findings highlight that PC is significantly associated with a number of risk factors for HIV infection. PC was associated with increased laboratory-confirmed STIs and reduced likelihood of using a condom in the last sexual intercourse. PC was also associated with lower intention of using or buying a condom in the next 3 months, increased fear of discussing condom use with sex partners, and more perceived barriers to negotiating condom use.

PC is often described in the context of abusive, power-imbalanced relationships.^14,36,37^ Consistent with the empirical literature, coercion was associated with physical and sexual abuse, and birth control sabotage. Whether in the context of an abusive relationship or not, male sex partners' reproductive desires may take precedence over women's reproductive choices, leading to unprotected sex and unwanted pregnancy. Male sex partner threats and lack of open communication exacerbate barriers to condom use and limit women's ability to enact condom use.^30,38^ Relational power imbalances have been shown to reduce women's ability to negotiate condom use or avoid unwanted sex.^39^ PC may be a related mechanism by which power imbalances and gender-based inequalities in sexual relationships translate into low rates of condom use that, in turn, heighten HIV/STI risk.

### Strengths and Limitations

No causal inference can be drawn from this analysis because of its cross-sectional nature. With the exception of STI diagnoses, other variables were self-reported and could, thus, be influenced by social desirability and recall bias. Furthermore, this study only included urban young African American women who were sexually active and who regularly consumed alcohol, therefore limiting the generalizability of the findings.

However, the inclusion of laboratory-confirmed STIs strengthens the observed association with PC. Furthermore, self-reported variables were assessed using audio computer-assisted self-interview to minimize, though not eliminate, information bias. Also, to further facilitate participants' recall and accurate reporting, variables were assessed over a relatively short time period, last 90 days. This timeline follow-back strategy has been demonstrated to provide reliable reports of HIV risk behaviors.^40,41^

### Conclusion

This study highlights that whereas it is critical to improve women's condom self-efficacy and negotiation skills, their ability to enact these skills may be limited given male partners' reproductive desires in the context of coercive relationships. As others have highlighted, HIV prevention interventions should take into consideration relationship power imbalances and contextual factors.^31^ A systematic review of sexuality and HIV education curricula found that programs that addressed gender and power were more effective in reducing STI rates than those that did not.^42^ Furthermore, programs that target both men and women to promote more equitable gender norms may effectively reduce interpersonal violence and improve reproductive health outcomes.^43^ The findings from the current analysis underscore the need for incorporating concepts of gender and power, while promoting gender-equitable social norms, in HIV prevention interventions. Finally, as one of the few studies focusing on African American women who regularly drink and are sexually active, this study constitutes a unique contribution to the literature on HIV/STI prevention needs among this understudied, vulnerable population.

## References

[1] Centers for Disease Control and Prevention. HIV Surveillance Report, 2016. Atlanta, GA: CDC; 2017.

[2] Centers for Disease Control and Prevention. HIV Surveillance Supplemental Report 2019. Estimated HIV Incidence and Prevalence in the United States, 2010–2016. Atlanta, GA: CDC; 2019.

[3] Centers for Disease Control and Prevention. STDs in Racial and Ethnic Minorities. 2018 Available at: https://www.cdc.gov/std/stats17/minorities.htm. Accessed March 29, 2019.

[4] Miller M Liao Y Gomez AM Factors associated with the prevalence and incidence of Trichomonas vaginalis infection among African American women in New York City who use drugs. J Infect Dis. 2008;197:503–509. 18275272 10.1086/526497

[5] Sorvillo F Smith L Kerndt P Trichomonas vaginalis, HIV, and african-Americans. Emerg Infect Dis. 2001;7:927–932. 11747718 10.3201/eid0706.010603 PMC2631893

[6] Cohen MS Classical sexually transmitted diseases drive the spread of HIV-1: back to the future. J Infect Dis. 2012;206:1–2. 22517911 10.1093/infdis/jis303

[7] Fleming DT Wasserheit JN From epidemiological synergy to public health policy and practice: the contribution of other sexually transmitted diseases to sexual transmission of HIV infection. Sex Transm Inf. 1999;75:3–17. 10.1136/sti.75.1.3 PMC1758168 10448335

[8] Kalichman SC Pellowski J Turner C Prevalence of sexually transmitted co-infections in people living with HIV/AIDS: systematic review with implications for using HIV treatments for prevention. Sex Transm Inf. 2011;87:183–190. 10.1136/sti.2010.047514 PMC4317792 21330572

[9] Cohen MS Sexually transmitted diseases enhance HIV transmission: no longer a hypothesis. Lancet. 1998;351:S5–S7. 10.1016/s0140-6736(98)90002-2 9652712

[10] Ward H Ronn M Contribution of sexually transmitted infections to the sexual transmission of HIV. Curr Opin HIV AIDS. 2010;5:305–310. 20543605 10.1097/COH.0b013e32833a8844 PMC2923028

[11] Sexton J Garnett G Rottingen JA Meta-analysis and meta-regression in interpreting study variability in the impact of sexually transmitted diseases on susceptibility to HIV infection. Sex Transm Dis. 2005;32:351–357. 15912081 10.1097/01.olq.0000154504.54686.d1

[12] van de Wijgert JH Morrison CS Brown J Disentangling contributions of reproductive tract infections to HIV acquisition in African Women. Sex Transm Dis. 2009;36:357–364. 19434010 10.1097/OLQ.0b013e3181a4f695

[13] DiClemente RJ Crittenden CP Rose E Psychosocial predictors of HIV-associated sexual behaviors and the efficacy of prevention interventions in adolescents at-risk for HIV infection: what works and what doesn't work? Psychosom Med. 2008;70:598–605. 18541908 10.1097/PSY.0b013e3181775edb

[14] Miller E Decker MR McCauley HL Pregnancy coercion, intimate partner violence and unintended pregnancy. Contraception. 2010;81:316–322. 20227548 10.1016/j.contraception.2009.12.004 PMC2896047

[15] Miller E Decker MR Reed E Male partner pregnancy-promoting behaviors and adolescent partner violence: findings from a qualitative study with adolescent females. Ambul Pediatr. 2007;7:360–366. 17870644 10.1016/j.ambp.2007.05.007

[16] Grace KT Anderson JC Reproductive coercion: a systematic review. Trauma Violence Abuse. 2018;19:371–390. 27535921 10.1177/1524838016663935 PMC5577387

[17] Miller E McCauley HL Tancredi DJ Recent reproductive coercion and unintended pregnancy among female family planning clients. Contraception. 2014;89:122–128. 24331859 10.1016/j.contraception.2013.10.011 PMC4018410

[18] Seth P DiClemente RJ Lovvorn AE State of the evidence: intimate partner violence and HIV/STI risk among adolescents. Curr HIV Res. 2013;11:528–535. 24476354 10.2174/1570162x12666140129103122

[19] Sales JM Monahan JL Brooks C Differences in sexual risk behaviors between lower and higher frequency alcohol-using African-American adolescent females. Curr HIV Res. 2014;12:276–281. 25053364 10.2174/1570162x12666140721122606 PMC4508002

[20] Curran TM Monahan JL Samp JA Sexual risk among African American women: psychological factors and the mediating role of social skills. Commun Q. 2016;64:536–552. 28490827 10.1080/01463373.2015.1132241 PMC5421988

[21] Van Der Pol B Ferrero DV Buck-Barrington L Multicenter evaluation of the BDProbeTec ET system for detection of chlamydia trachomatis and neisseria gonorrhea in urine specimens, female endocervical swabs, and male urethral swabs. J Clin Microbiol. 2001;37:1008–1016. 10.1128/JCM.39.3.1008-1016.2001 PMC87865 11230419

[22] Schmiege SJ Broaddus MR Levin M Randomized trial of group interventions to reduce HIV/STD risk and change theoretical mediators among detained adolescents. J Consult Clin Psychol. 2009;77:38–50. 19170452 10.1037/a0014513 PMC9017688

[23] Wingood GM DiClemente RJ Partner influences and gender-related factors associated with noncondom use among young adult African American women. Am J Community Psychol. 1998;26:29–51. 9574497 10.1023/a:1021830023545

[24] DiClemente RJ Wingood GM Harrington KF Efficacy of an HIV prevention intervention for African American adolescent girls: a randomized controlled trial. JAMA. 2004;292:171–179. 15249566 10.1001/jama.292.2.171

[25] Williams CT Latkin CA The role of depressive symptoms in predicting sex with multiple and high-risk partners. J Acquir Immune Defic Syndr. 2005;38:69–73. 15608528 10.1097/00126334-200501010-00013

[26] Melchior LA Huba GJ Brown VB A short depression index for women. Educ Psychol Meas. 1993;53:1117–1125.

[27] Campbell JC Health consequences of intimate partner violence. Lancet. 2002;359:1331–1336. 11965295 10.1016/S0140-6736(02)08336-8

[28] Ellsberg M Jansen HA Heise L Intimate partner violence and women's physical and mental health in the WHO multi-country study on women's health and domestic violence: an observational study. Lancet. 2008;371:1165–1172. 18395577 10.1016/S0140-6736(08)60522-X

[29] Gonzalez-Guarda RM Williams JR Williams W Determinants of HIV and sexually transmitted infection testing and acquisition among female victims of intimate partner violence. J Interpers Violence. 2019:886260519827662. 30755076 10.1177/0886260519827662 PMC6692236

[30] Kalichman SC Williams EA Cherry C Sexual coercion, domestic violence, and negotiating condom use among low-income African American women. J Womens Health. 1998;7:371–378. 9580917 10.1089/jwh.1998.7.371

[31] Seth P Wingood GM Robinson LS Abuse impedes prevention: the intersection of intimate partner violence and HIV/STI risk among young African American women. AIDS Behav. 2015;19:1438–1445. 25399033 10.1007/s10461-014-0940-7 PMC4433610

[32] Garcia-Moreno C Jansen HA Ellsberg M Prevalence of intimate partner violence: findings from the WHO multi-country study on women's health and domestic violence. Lancet. 2006;368:1260–1269. 17027732 10.1016/S0140-6736(06)69523-8

[33] Pflieger J Cook E Niccolai L Racial/ethnic differences in patterns of sexual risk behavior and rates of sexually transmitted infections among female young adults. Am J Public Health. 2013;103:903–909. 23488501 10.2105/AJPH.2012.301005 PMC3698802

[34] Willie T Kershaw T Campbell JC Intimate partner violence and PrEP acceptability among low-income, young black women: exploring the mediating role of reproductive coercion. AIDS Behav. 2017;21:2261–2269. 28409266 10.1007/s10461-017-1767-9 PMC5823269

[35] Sutherland MA Fantasia HC Fontenot H Reproductive coercion and partner violence among college women. J Obstet Gynecol Neonatal Nurs. 2015;44:218–227. 10.1111/1552-6909.12550 25656602

[36] Barber JS Kusunoki Y Gatny H The dynamics of intimate partner violence and the risk of pregnancy during the transition to adulthood. Am Sociol Rev. 2018;83:1020–1047. 30739942 10.1177/0003122418795856 PMC6364682

[37] Clark LE Allen RH Goyal V Reproductive coercion and co-occurring intimate partner violence in obstetrics and gynecology patients. Am J Obstet Gynecol. 2014;210:42 e41–48. 24055583 10.1016/j.ajog.2013.09.019

[38] Swan H O'Connell DJ The impact of intimate partner violence on women's condom negotiation efficacy. J Interpers Violence. 2012;27:775–792. 21987514 10.1177/0886260511423240 PMC4451787

[39] Crosby RA DiClemente RJ Wingood GM Sexual agency versus relational factors: a study of condom use antecedents among high-risk young African American women. Sex Health. 2008;5:41–47. 18361853 10.1071/sh07046

[40] Weinhardt LS Carey MP Maisto SA Reliability of the timeline follow-back sexual behavior interview. Ann Behav Med. 1998;20:25–30. 9755348 10.1007/BF02893805 PMC2435070

[41] Janssen T Braciszewski JM Vose-O'Neal A A comparison of long- vs. short-term recall of substance use and HIV risk behaviors. J Stud Alcohol Drugs. 2017;78:463–467. 28499115 10.15288/jsad.2017.78.463 PMC5440371

[42] Haberland NA The case for addressing gender and power in sexuality and HIV education: a comprehensive review of evaluation studies. Int Perspect Sex Reprod Health. 2015;41:31–42. 25856235 10.1363/4103115

[43] Jewkes R Flood M Lang J From work with men and boys to changes of social norms and reduction of inequities in gender relations: a conceptual shift in prevention of violence against women and girls. Lancet. 2015;385:1580–1589. 25467578 10.1016/S0140-6736(14)61683-4

